# Therapeutic Targeting of Retinal Immune Microenvironment With CSF-1 Receptor Antibody Promotes Visual Function Recovery After Ischemic Optic Neuropathy

**DOI:** 10.3389/fimmu.2020.585918

**Published:** 2020-11-13

**Authors:** Yizhen Tang, Zebin Xiao, Li Pan, Dongli Zhuang, Kin-Sang Cho, Kyle Robert, Xiaoxiao Chen, Lian Shu, Guangxian Tang, Jihong Wu, Xinghuai Sun, Dong F. Chen

**Affiliations:** ^1^ Eye Institute and Department of Ophthalmology, Eye & ENT Hospital, Fudan University, Shanghai, China; ^2^ Schepens Eye Research Institute of Massachusetts Eye and Ear, Department of Ophthalmology, Harvard Medical School, Boston, MA, United States; ^3^ NHC Key Laboratory of Myopia, Fudan University, Shanghai, China; ^4^ Shanghai Key Laboratory of Visual Impairment and Restoration, Fudan University, Shanghai, China; ^5^ Department of Radiology, Eye & ENT Hospital, Fudan University, Shanghai, China; ^6^ School of Optometry, The Hong Kong Polytechnic University, Hong Kong, Hong Kong; ^7^ Department of Ophthalmology, 1st Hospital of Shijiazhuang, Shijiazhuang, China

**Keywords:** visual acuity, retinal ganglion cell, neurodegeneration, retinal ischemia/reperfusion, colony stimulating factor-1, microglia, positive scotopic threshold response

## Abstract

Retinal ischemia/reperfusion injury (RI) is a common cause of irreversible visual impairment and blindness in elderly and critical unmet medical need. While no effective treatment is available for RI, microglial activation and local immune responses in the retina are thought to play important roles in the pathophysiology of neurodegeneration. While survival and activation of microglia depend critically on colony-stimulating factor receptor (CSF-1R) signaling, it remains unclear if targeting the retinal immune microenvironments by CSF-1RAb after RI is sufficient to rescue vision and present a potentially effective therapy. Here we used rodent models of RI and showed that retinal ischemia induced by acute elevation of intraocular pressure triggered an early activation of microglia and macrophages in the retina within 12 h. This was followed by lymphocyte infiltration and increased production of pro-inflammatory cytokines. Intravitreal injection of CSF-1R neutralizing antibody (CSF-1RAb) after RI significantly blocked microglial activation and the subsequent T cell recruitment. This also led to improved retinal ganglion cell survival and function measured by cell quantification and electroretinogram positive scotopic threshold responses, as well as increased visual acuity and contrast sensitivity as assessed by optomotor reflex-based assays, when compared to the isotype-treated control group. Moreover, the administration of CSF-1RAb efficiently attenuated inflammatory responses and activation of human microglia in culture, suggesting a therapeutic target with human relevance. These results, together with the existing clinical safety profiles, support that CSF-1RAb may present a promising therapeutic avenue for RI, a currently untreatable condition, by targeting microglia and the immune microenvironment in the retina to facilitate neural survival and visual function recovery.

## Introduction

Ischemia is a broad term to describe a restriction of blood supply to tissues and a key contributing factor to neural damage in the brain and retina, such as that resulted from stroke, diabetes, diabetic retinopathy, glaucoma, etc. Acute retinal ischemia, including central artery or vein occlusions, is a stroke-equivalent most common cause of irreversible vision loss in elderly (1). It induces damage to the inner retina and permanent loss of retinal ganglion cells (RGCs) (2, 3). Currently, no effective treatment is available for acute retinal ischemia in clinic, and the underlying mechanisms of reperfusion-induced retinal neuron injury is not fully understood.

Evidence has been shown that retinal ischemia/reperfusion injury (RI) is accompanied by glial activation, neuroinflammation, and induction of a cytokine cascade (2, 3). Microglia are the residential immune competent cells colonized in the central nervous system (CNS) and essential in maintaining the neuro-retinal homeostasis and innate immune defense mechanisms (4, 5). Resting microglia typically present a highly ramified morphology (6) and contribute to retinal homeostasis by performing immune surveillance functions (7, 8). Virtually, under all insults or disease conditions microglia become activated and undergo phenotypic and functional changes, which represents a fundamental innate immune mechanism to protect the retina from infection or injury. However, uncontrolled microglial activation is detrimental and leads to neurodegeneration and retinal cell death (9). Microglial activation is associated with increased production of inflammatory cytokines and oxidative stress, as well as subsequent infiltration of circulating immune cells (10); they are thought to contribute to the primary early events occurring before neuron death (11, 12). Given to the importance of microglial activation in ischemia-induced neural pathology, they represent an attractive target for therapeutic interventions.

Colony-stimulating factor-1(CSF-1) is a growth factor essentially involved in the proliferation, differentiation, and survival of microglia *via* CSF-1 receptor (CSF-1R) (13, 14). Importantly, evidence suggests that CSF-1R signaling is also critical in mediating microglial activation after disease or injury, as short-term administration of CSF-1R inhibitor following neural injury reduced neurodegeneration and neurological deficits (9) and attenuated the disease progression (6). Nevertheless, long-term depletion of CSF-1R resulted in microglial elimination from the CNS (15–18), and administration of CSF-1R antagonists over 7 consecutive days reversibly depleted 90% microglia (19), leading to the increased infarct size after stroke (20). In addition, depletion of microglia in the retina exaggerated retinal ganglion cell (RGC) loss following excitotoxin damage (21). Thus, it remains controversial if administration of CSF-1R antagonist or neutralizing antibody (CSF-1RAb) represents a therapeutic strategy to suppress microglial activation and prevent excessive immune responses.

In the present study, we proposed to test if targeting CSF-1R after RI suppressed microglial activation, changed the immune microenvironment in the retina. Our data showed that intravitreal injection of CSF-1RAb after RI attenuated retinal ganglion cell degeneration and vision loss through countering microglial activation and retinal immune responses, rather than depleting the microglia. These results establish the translational potential of CSF-1RAb for treating RI.

## Materials and Methods

### Animals

Male Sprague-Dawley rats weighing 200–250 g (8 weeks old, from SLAC Laboratory Animal Company, Shanghai, China) and C57BL/6J wild-type mice (8–10 weeks old, from Jackson Laboratories, Bar Harbor, ME, USA) were housed under a 12 h light/dark cycle and kept under pathogen-free conditions. All animals were handled in accordance with the National Institute of Health and the Association for Research in Vision and Ophthalmology, and all experimental procedures and the use of animals were approved and monitored by the Animal Care Committee of the Eye and ENT Hospital of Fudan University and Schepens Eye Research Institute of Massachusetts Eye and Ear. Animals were selected randomized for treatment and observed blinded.

### Acute Retinal Ischemia/Reperfusion Injury

Retinal ischemia/reperfusion injury (RI) was induced in mouse and rat as previously described (22–24). Briefly, animals were anesthetized with a mixture of 120 mg/kg ketamine and 20 mg/kg xylazine in sterile saline (1:1:6). Retinal ischemia was induced unilaterally in the right eye. One percent tropicamide (Bausch & Lomb Inc., Tampa, FL, USA) and 0.5% proparacaine hydrochloride (Bausch & Lomb Inc., Tampa, FL, USA) were applied topically onto the cornea for pupil dilation and topical anaesthetization. The cornea was gently punctured using a 30-gauge needle to establish an access for a glass micropipette connected to an intravenous tube set (Abbott Laboratories, North Chicago, IL, USA) with sterile physiological saline reservoir (0.9% sodium chloride, Hospira, Inc., Lake Forest, IL, USA) at a vertical distance of 115 cm above the eye in mice and 150 cm above the eye in rats, that led to acute elevation of intraocular pressure (IOP) to 85 mmHg in mice and 110 mmHg in rats, respectively. Whitening of the fundus observed under the microscope was regarded as a sign of successful induction of retinal ischemia. After 60 min of acute IOP elevation, the saline reservoir was slowly lowered, and the needle was removed from the anterior chamber. Vessel reperfusion in the fundus was observed under the microscope to ensure the reperfusion of the retina. In the control group, the right cornea was punctured without saline reservoir raised above and without IOP elevation. Animals were sacrificed at 12 h, day 1, day 2, day 7 (7d), day 14 (14d), and day 28 (28d), respectively, after retinal ischemic injury.

### Flow Cytometry

To detect the dynamic changes of retinal microglia, macrophage and lymphocyte infiltration, and T cell responses following acute RI, animals were transcardially perfused with saline. Retinas were dissected and digested in papain for 15 min in 37°C and stopped by the ovomucoid protease inhibitor. After filtration through a 70 µm nylon cell strainer (Corning, USA, Cat#431751), cell numbers are counted. Cells were then washed in IsoFlow (Beckman Coulter Inc, Brea, CA, USA, Cat#50169F) and reacted with following primary antibodies: APC-conjugated anti-rat CD11b antibody (IgA, Clone WT.5, BD biosciences, Franklin Lakes, NJ, USA, Cat#562102), PE-CY7-conjugated anti-rat CD45 antibody (IgG1, Clone OX-1, BD biosciences, Franklin Lakes, NJ, USA, Cat#561588), Pacific Blue-conjugated anti-mouse CD11b antibody (IgG2b, Clone M1/70, BioLegend, San Diego, CA, USA, Cat#101224), PE-CY7-conjugated anti-mouse CD45 antibody (IgG2b, Clone 30-F11, BD biosciences, Franklin Lakes, NJ, USA, Cat#552848). For Ki67 detection, dissociated retinal cells were permeabilized with Perm/Wash buffer and stained with Alexa Flour 488-conjugated anti-rat Ki67 antibody (IgG, Cell Signaling Technology, MA, USA, Cat#11882S). To analyze T cells in the superior cervical lymph nodes (LNs) (25), superior cervical LNs were collected and cells were mechanically dissociated with two forceps. After filtration to remove cell clumps and cell counts, dissociated cells were rinsed with IsoFlow (Beckman Coulter Inc, Brea, CA, USA, Cat#50169F) and reacted with FITC-conjugated anti-mouse CD4 antibody (IgG2b, clone GK1.5, BioLegend, San Diego, CA, USA, Cat#100406). Thereafter, cells were permeabilized with Perm/Wash buffer (Thermo Fisher, USA, Cat#00552300) and labeled with PE-labeled anti-mouse IFN-γ antibody (IgG1, clone XMG1.2, BioLegend, San Diego, CA, USA, Cat#505808), for detection of Th1 cells. A four-laser Becton- Dickinson FACS Calibur (Beckman Coulter, USA) and BD LSR II Flow Cytometer (BD Biosciences) was used to collect the data, and data analysis were carried out using Flowjo (FlowJo LLC, Ashland, OR, USA).

### Immunofluorescence Labeling

RGC and microglia was immunolabeled in retinal flat-mounts using a standard protocol as previously described (26). To prepare for the flat-mounts, eyeballs were fixed in 4% PFA for 1–3 h at room temperature. The retina was dissected and washed in phosphate buffered saline (PBS). After incubated in a blocking solution containing 10% goat serum, 3% donkey serum, 1% Bovine Serum Albumin, 0.5% triton, and 0.5% Tween for 3h, retinal flat-mounts were incubated with a primary antibody against an RGC specific-marker, Brn3a (27, 28) (1:500, Millipore, Darmstadt, Germany, Cat# MAB1585) and a microglia marker Iba-1 (1:200, Wako, Japan, Cat# 019-19741) and astroglia marker glial fibrillary acidic protein (GFAP, 1:200, Abcam, USA, Cat#ab53554) for overnight. This were followed by incubation with a Cy3-conjugated donkey-anti-mouse secondary antibody (1:400, Jackson ImmunoResearch Inc, West Grove, PA, USA, Cat# 715-225-150), Cy2-conjugated donkey-anti-rabbit secondary antibody (1:400, Jackson ImmunoResearch Inc, West Grove, PA, USA, Cat#711-165-152), and Cy5-conjugated donkey-anti-goat secondary antibody (1:400, Jackson ImmunoResearch Inc, West Grove, PA, USA, Cat# 705-175-147). For cell quantification, retinal flat-mounts were divided into four areas including superior, temporal, nasal and inferior. Four standard regions from the peripheral to central retina were selected as previously described (26). Total 16 single slice confocal images were collected from each retina at 400× magnification (Leica TCS-SP8). The numbers of RGCs and microglia, microglia dendrite length, circularity and area of microglia cell body were quantified. Immunointensity of GFAP and CD68 were quantified by outlining the cells and measuring the integrated intensity. All quantification procedures were conducted *via* Image J by two researchers in a masked fashion.

### qPCR Detection

Total RNA was extracted from the retina and the cells using Quick-RNA Microprep Kit (Zymo Research, Cat#R1051) according to the manufacturer’s protocol. The mRNA was then converted to cDNA using a SuperScript IV First-Strand Synthesis System (Takara Biotechnology, USA, Cat#RR036A) according to the manufacturer’s instructions. Quantitative polymerase chain reaction (qPCR) analysis was performed using SYBR Green I dye (Takara Biotechnology, USA, Cat#RR420) and KAPA SYBR FAST kit (Kapa Biosystems, USA, Cat#07959397001). All primers were synthesized by Sangon Biotech (Shanghai, China) and Integrated DNA Technologies (USA). The sequences of all primers are listed in **Table S1**. The relative mRNA levels were normalized to housekeeping gene glyceraldehyde 3-phosphate dehydrogenase (GAPDH) levels. Expression was analyzed using the 2-ΔΔCt method.

### Intravitreal Injections

Intravitreal injection was performed as previously described (29). CSF-1RAb, isotype control, or sterile saline was injected intravitreally on days 1, 7, 14, and 21 following acute retinal ischemia. A glass micropipette was connected to a Hamilton syringe to ensure the small volume (2 µl) of intravitreal injection. A 30-gauge needle was used to gently puncture the site posterior to the limbus to enable an entry site for a glass micropipette in the right eye. Two microliter of 0.5 mg/ml of ligand blocking antibody anti-mouse CSF-1R monoclonal antibody (IgG2a, Clone AFS98, Thermo Fisher, USA, Cat#16115282) (30, 31) or isotype control (IgG2a, Clone eBR2a, Thermo Fisher, USA, Cat#16432182) was injected intravitreally through a glass micropipette. The same entry site was used during the repeated injections to ensure minimal injury to the eye unless vessel growth was noted around the entry site. No apparent signs of inflammation or degeneration was observed after intravitreal injections.

### Electroretinography and Positive Scotopic Threshold Response

Positive scotopic threshold response (pSTR) of electroretinogram (ERG) is an established surrogate measure for RGC functions (32). Animals were dark adapted for at least 6 h before recordings. All procedures were performed in a dark room with red safety light. Mice were anesthetized with 120 mg/kg Ketamine and 20 mg/kg Xylazine. One percent tropicamide (Bausch & Lomb Inc., Tampa, FL, USA) was applied for pupil dilation. Mice were then placed in the 37°C warm pad in a Ganzfield bowl while recording to prevent hypothermia. Gold wire ring electrodes were placed on both corneas, the reference and ground electrodes were inserted subcutaneously in the mid-frontal head area and back area near the tail. GenTeal lubricating gel (Alcon, Fort Worth, TX, USA) was applied on both corneas to keep moisture. ERG was recorded in both eyes simultaneously. Under the scotopic conditions, pSTR was detected and obtained with flash intensities at 6.57E-5 cd.s/m^2^ and 1.7E-4 cd.s/m^2^ by an average of 40 responses per intensities. Data were processed by the software included in the ERG recorder system (Espion Electroretinography System; Diagnosys LLC, Lowell, MA, USA). The pSTR was measured from the baseline to the peak of the positive deflection.

### Optomotor Response/Reflex (OMR)-Based Visual Assessment

The setup of the OMR assay was established as previously described (33). Briefly, mice were placed on a platform located in the center of an enclosure box composed by four identical 17-inch liquid crystal display monitors presenting moving black and white gratings (**Figure 5C**). To ensure ventilation, whisper fans were set behind the monitors. Mouse was allowed to move freely on the platform when exposed to the visual stimulation presented on the surrounding monitors. The vertical sine-wave gratings rotated at a constant speed (12°/s) on the monitors. The direction of rotation (clockwise and counterclockwise) was alternated. When detecting the stimulus presented on the monitors, the mouse stopped moving and began headtracking, which could be observed as a smooth reflexive head movement based on the speed and direction of the rotation. To ensure accuracy, two experienced investigators scored OMR responses in a masked fashion. At least three times of head tracking observed by both investigators were required to conclude the detection of the stimulus. If the mouse happened to slip or jump off the platform during testing, it was returned to the platform and the test was resumed until the mouse calmed down. Spatial frequency thresholds were determined by the staircase paradigm. The visual acuity (VA) threshold was detected by setting sinusoidal gratings at 100% contrast (black stripe, 0.3 cd/m^2^; white stripe, 205 cd/m^2^). As mouse reached the threshold of spatial frequency detectability, tracking reflex responses to the moving gratings was no longer detectable. The highest spatial frequency that reflex response was detected from a mouse was determined to be the final VA of the mouse. The contrast sensitivity (CS) threshold was measured by setting sinusoidal gratings at a fixed spatial frequency. As the contrast reduced, the mouse ceased to track the gratings and the threshold was detected, which was presented as Michelson contrast according to the brightness of the screen (maximum-minimum)/(maximum+minimum). The reciprocal value of the threshold which was determined at five spatial frequencies (0.067, 0.133, 0.209, 0.266, 0.4 cycle per degree [cyc/deg]) was finally calculated as the CS.

### Microglial Cultures

Primary microglia were isolated from C57BL/6J mouse pups aged postnatal day 1–5 after euthanasia, as previously described (34, 35). The cortex of the brain was cut and centrifuged in Minimal Essential Medium (MEM, Gibco, USA, Cat#11885-084) and resuspended in fresh MEM with 10% fetal bovine serum (FBS, Sigma, USA, Cat#F0926). Cells were seeded in 75 cm^2^ flasks (Corning, USA, Cat#353136) and incubated at 37°C with 5% CO_2_. The medium was changed 48 h later to remove cellular debris. After 10–14 days of incubation, culture flasks were placed on a shaker, shaking at a speed of 65 rpm for 5 h in the incubator. After most microglia had detached, the culture media was carefully aspirated, collected, and centrifuged and cells were resuspended in fresh MEM. Microglia were seeded onto 12-well plate and settled for 3–5 days before experiments. The medium was changed three times a week. Microglia were stimulated with lipopolysaccharide (LPS,100 ng/ml, Sigma, USA, Cat#L6529-1MG) and adenosine triphosphate (ATP, 100 μM, EMD Millipore, USA, Cat# 5.05734.0001) to induce inflammatory response. Various doses of CSF-1RAb (1, 5, 10, 50 μg/ml) were added to the culture medium after LPS and ATP stimulation. Isotype IgG was served as a control. Microglia were harvested 24 h after stimulation for RNA extraction and qPCR analysis.

Human microglia cell line, HMC3 (ATCC, USA, Cat#CRL-3304), was purchased from ATCC and cells were cultured in Eagle’s Minimum Essential Medium (EMEM, ATCC, Cat#30–2003™) supplemented with 10% FBS (FBS, ATCC, Cat#30–2020™). Cells were plated in T75 flasks (Corning, USA, Cat#353136) and incubated at 37°C with 5% CO2. The medium was changed every 48 h. Cells were then seeded onto 24-well plates and stimulated with LPS and ATP as previously described (36). Various doses of CSF-1RAb (1, 5, 10, and 50 μg/ml) were added to the culture media immediately following LPS and ATP stimulation. Cells were harvested 24 h after for qPCR analysis.

### Proteome Profiler Mouse Cytokine Array

The Proteome Profiler Mouse Cytokine Array Kit (Panel A; R&D Systems, USA) was used to analyze the cytokine arrays using filtered supernatant collected from primary mouse microglial cultures at 48 h incubation in the presence or absence of CSF-1RAb LPS and ATP. Assays were carried out according to the manufacturer’s protocol as previously described (37). The array membranes were imaged with film developer (SRX-101A, Konica Minolta, USA) and the results were analyzed using ImageJ software.

### Statistical Analysis

Statistics were analyzed using GraphPad Prism 6 software (GraphPad Inc., La Jolla, CA, USA). Increasing evidence suggests that retinal ischemia induces systemic immune responses, which can potentially affect cellular responses of the contralateral eye; therefore, different groups of animals, rather than the contralateral eye, were used as controls. Statistical differences among time points and doses were made by One-way ANOVA and Dunnett’s multiple comparisons test. Statistical differences among treatment groups were made by Two-way ANOVA and Tukey’s multiple comparisons test. Probability value of *P* < 0.05 were considered to be statistically significant. Data were expressed as mean ± S.E.M.

## Results

### Retinal Reperfusion Injury Induced Early Activation and Proliferation of Microglia

**Figure 1:**
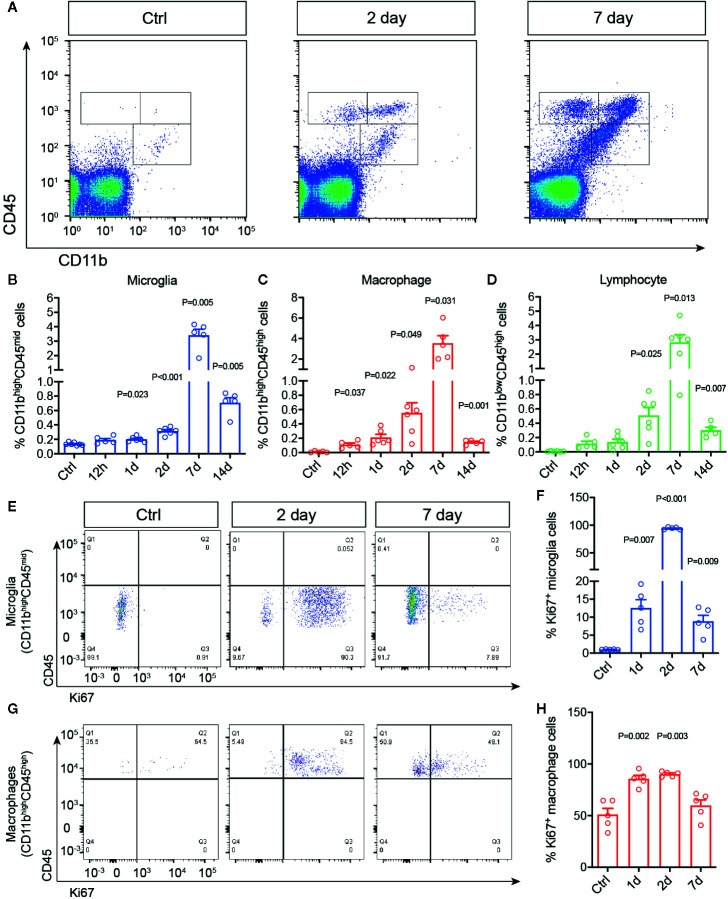
Retinal ischemia/reperfusion injury induced early changes in retinal immune microenvironment. **(A)** Representative plots of flow cytometry that detected cells immunolabeled for CD45 and CD11b in retinas of a control mouse (Ctrl) or mice at 2 (Day 2) and 7 days (Day 7) post RI. **(B–D)** Percentages of retinal microglia **(B)**, macrophages **(C)** and lymphocytes **(D)** in control (Ctrl) and 12 h–14 d post RI (n ≥ 5/group). **(E–H)** Representative plots of flow cytometry **(E, G)** and quantification **(F, H)** of Ki67^+^ cells gated on retinal microglia (CD11b^high^CD45^mid^) **(F)** and macrophages (CD11b^high^CD45^high^) **(H)**. Note that control group is a cumulative value for all timepoints. One-way ANOVA compared to control (n = 5/group).

To study the chronological changes of retinal immune microenvironment induced by RI, we investigated dynamics of immune cell activation and infiltration in the rat retinas. Adult SD rats were subjected to acute elevation of intraocular pressure to 110 mmHg for 1 h (38). Microglial activation and infiltration of macrophages and lymphocytes were quantitatively assessed by flow cytometry at 12 h to 14 days post injury. Primary antibody against CD45 was used to identify all leukocytes, as it distinguishes infiltrated macrophages by their expression of a high level CD45 (CD45^high^), from microglia, which express a moderate level of CD45 (CD45^mid^). In addition, CD11b was used to separate macrophage (CD11b^high^CD45^high^) and microglia (CD11b^high^CD45^mid^) from infiltrated lymphocytes, which are CD11b negative (CD11b^low^CD45^high^) (39, 40) (**Figure 1A**). In the control untreated retinas, as expected only resting microglia were detected (**Figures 1B–D**). Retinal ischemia/reperfusion injury led to significant increases of microglia and infiltrated macrophages at as early as 12 h post injury (*P* = 0.047), suggesting an early induction of retinal immune responses. The numbers of microglia and macrophages reached a peak of a 6–8 folds increase over the controls at day 7 after RI. Lymphocyte infiltration (CD11b^low^CD45^high^) was detectable beginning at day 2 (*P* = 0.025) post injury while it also reached a peak by day 7. Thus, 1 h acute RI induced a prolonged phase of retinal immune responses involving activation of microglia and infiltrations of macrophages and lymphocytes which peaked on day 7 after RI.

We then asked if RI also triggers microglial and macrophage proliferation in the retina using Ki67 immunolabeling. Ki67 is a commonly used marker for detecting proliferating cells at all active phases of the cell cycle (G_1_, S, G_2_, M), except in the resting phase (G_0_) (41). In the untreated retinas, few Ki67+ cells were detected by flow cytometry. In contrast, RI induced drastic increase of CD11b^high^CD45^mid^Ki67+ cells, suggesting microglia proliferation, within 24 h after RI (*P* = 0.007), and it quickly reached a peak on day 2 but decreased after day 7 (**Figures 1E, F**). A moderate increase of CD11b^high^CD45^high^Ki67+ cells or proliferating macrophages was also noted between days 1–7 (**Figures 1G, H**). These data suggest that RI induced early immune responses and robust proliferation of microglia after injury.

### Retinal Ischemia Induced Pro-Inflammatory Microglial and Macrophage Responses

**Figure 2:**
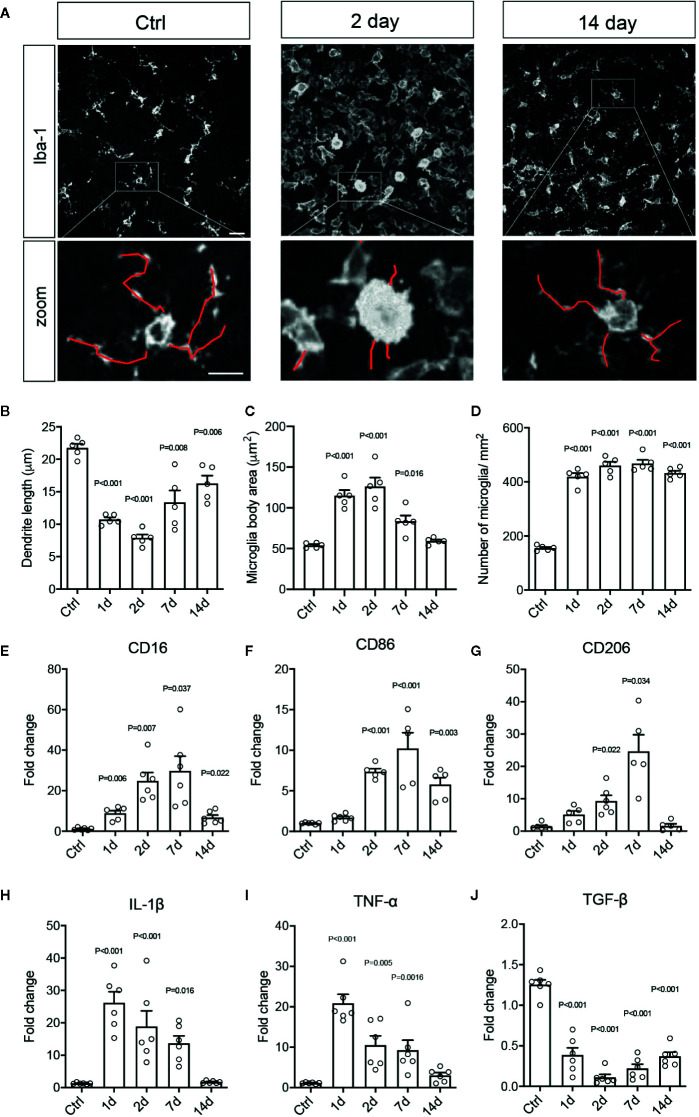
Retinal ischemia/reperfusion injury induced retinal microglial and macrophage activation and inflammation. **(A)** Epifluorescence photomicrographs of Iba1-immunolabeled cells from retinal flat-mounts taken from a control mouse (Ctrl) and mice at days 2 (Day 2) and 7 (Day 7) post RI. Insets showing enlarged images of Iba-1^+^ cells. Scale bar: 50 μm; 25 μm (inset). **(B–D)** Quantification of dendritic length **(B)**, cell body areas **(C),** and cell densities **(D)** of Iba-1^+^ cells in retinal flat-mounts of control mice (Ctrl) and mice at 1-14 days post RI. **(E–J)** Results of qPCR showing fold changes in mRNA levels of CD16 **(E)**, CD86 **(F)**, CD206 **(G)**, IL-1β **(H),** TNF-α **(I)**, and TGF-β **(J)** in retinas of control mice and mice at days 1–14 post RI. Note that control group is a cumulative value for all timepoints. One-way ANOVA compared to control (n ≥ 5/group).

Activated microglia/macrophages are thought to undergo signature morphological changes, upregulate specific activation markers, including CD16, CD86, and CD206, and release pro-inflammatory cytokines, such as tumor necrosis factor-α (TNF-α) and interleukin-1β (IL-1β). To further characterize the phenotypes of microglia and infiltrated macrophages after RI, we quantified the morphological differences of Iba-1 immunolabeled cells, which reacts with both microglia and macrophages. These included dendritic lengths, body area, and number of microglia in retinal flat-mounts. In control retinas, most Iba-1^+^ cells were resting microglia displaying ramified cell morphology; whereas, RI induced a large increase in Iba-1^+^ cells within a day, and many exhibited amoeba-like morphology, larger cell bodies and shorter dendrites (**Figures 2A–D**). Results of quantitative PCR (qPCR) confirmed the significant upregulation of activated microglial markers, CD16, CD86, and CD206, which were peaked on day 7 after RI (*P* < 0.001) (**Figures 2E–G**). Moreover, significant induction of pro-inflammatory cytokines, TNF-α, IL-1β, IL-6, and IFN-γ and downregulation of anti-inflammatory cytokine TGF-β (**Figure 2J** and **Figure S1**) were also detected post RI. These results further support that RI induced pro-inflammatory responses in the retina.

### Therapeutic Administration of Anti-CSF1R Attenuated RI-Induced Immune Responses

**Figure 3:**
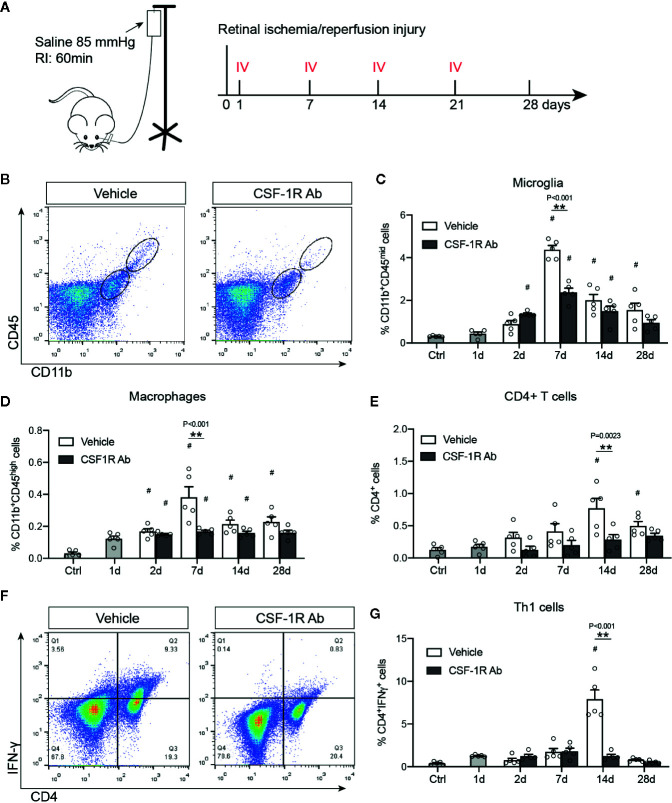
Targeting microglia by CSF-1R Ab attenuated retinal immune responses after RI. **(A)** Schematic illustration of the RI model and intravitreal injection (IV) schedules. **(B)** Representative plots of flow cytometry studying CD45 and CD11b double-immunolabeled retinal cells taken from mice received isotype- (Vehicle) or CSF-1RAb injection at days 7 post RI. (C-E) Quantifications of microglia (CD11bhighCD45mid ) **(C)**, macrophages (CD11bhighCD45high) **(D)** and CD4^+^ T cells **(E)** in the retinas of control (Ctrl), isotype- (Vehicle) and CSF-1RAb-injected mice at days 1 – 28 post RI. Retinal cells were double-immunolabeled for CD45 and CD11b. Note that mice started to receive saline- or CSF-1RAb-injection day 1 post RI. **(F)** Representative plots of flow cytometry showing cells from the superior cervical lymph nodes of mice received isotype- (Vehicle) or CSF-1RAb injection at days 7 post RI. Cells were double-immunolabeled for CD4 and IFN-γ. **(G)** Percentage of IFN-γ+CD4+ cells in lymph nodes by flow cytometry. Two-way ANOVA, *P < 0.05, **P < 0.01 comparing between vehicle and CSF-1RAb group, ^#^ *P* < 0.05 vs compared to control (n ≥5/group).

Microglia depends on CSF-1R signaling to proliferate and survive (13). As microglial proliferation and activation are involved early in RI-induced pathological changes, we sought to investigate if CSF-1R may represent an attractive target for therapeutic interventions. The rat anti-mouse CSF-1R (AFS98, CSF-1RAb) was reported to effectively block CSF-1R signaling and ligand binding (30, 31). We thus compared mice that received weekly therapeutic administrations of CSF-1RAb or saline (vehicle control) *via* intravitreal injection, starting at day 1 post RI and injected weekly for a total of 4 weeks (**Figure 3A**). Adult C57BL/6J mice were subjected to acute elevation of intraocular pressure to 85 mmHg for 60 min (**Figure 3A**). Dynamic changes of microglial activation and macrophage infiltration in mice after RI were verified using the vehicle control group. Analysis with flow cytometry using CD11b and CD45 double-immunolabeling revealed an early increase in numbers of microglia (CD11b^high^CD45^mid^) and macrophages (CD11b^high^CD45^high^) that were peaked on day 7 post RI in mice (**Figures 3B–D**), similar to that were seen in rats. We previously showed that RI also induced retinal infiltration of lymphocytes, particularly CD4^+^ IFN-γ-producing T helper (Th1) cells, which participate in the propagation of RGC damage (24). We confirmed that CD4^+^ T cell infiltration into the retina was peaked on day 14 after RI (*P* = 0.021) (**Figure 3E**), a week later after microglial and macrophage activations reached their peaks. In CSF-1R-Ab injected mice, we observed significantly reduced numbers of microglia and infiltrated macrophages compared to vehicle-treated group (**Figures 3B–D**), as well as attenuated CD4^+^ T cell infiltration into the retina (**Figure 3E**). They support that early activation of microglia and macrophages contribute critically to the initiation of CD4^+^ T cell responses post RI. To corroborate the finding that CSF-1RAb attenuated RI-induced T cell responses, we analyzed the number of Th1 (IFN-γ^+^CD4^+^) T cells in the draining lymph nodes of the retina—the superior cervical lymph nodes. In agreement with the above finding, significant increase of Th1 cells was noted in the draining lymph nodes of vehicle control group compared to non-injected control mice, and this was again attenuated in CSF-1RAb-treated mice (**Figures 3F, G**).

**Figure 4:**
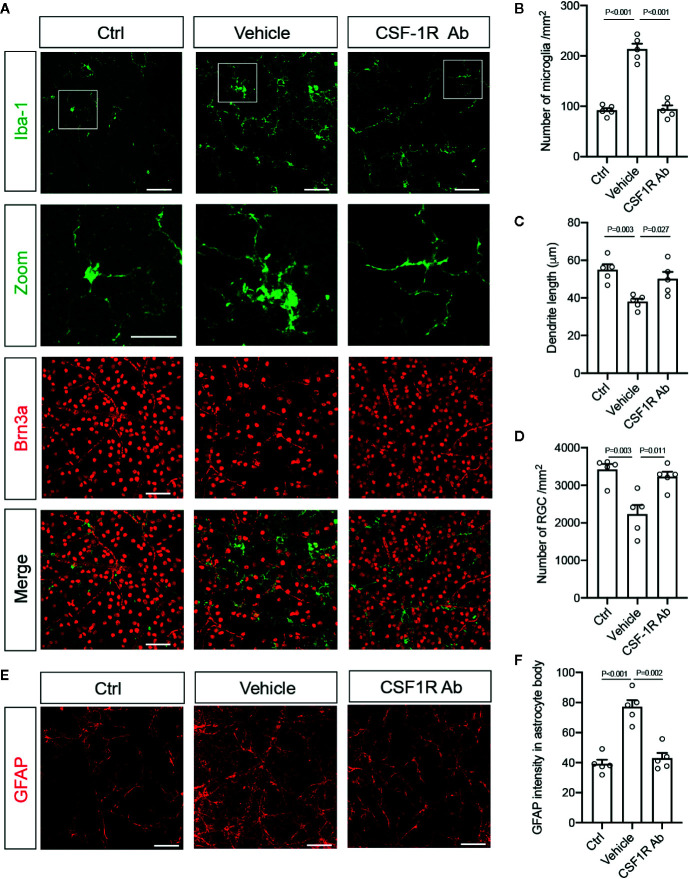
Targeting microglia by CSF-1RAb attenuated morphological changes of microglia, improved RGC survival, and suppressed reactive gliosis after RI. **(A)** Epifluorescent photomicrographs of retinal flat-mounts from mice at 4w after RI and double-immunolabeled for Iba-1 (green) and Brn3a (red). Scale bar: 50 μm, 25 μm (inset). **(B–D)** Quantification of Iba-1^+^ cells **(B)**, dendritic lengths of Iba-1^+^ cells **(C)**, and RGC densities **(D)**. **(E)** Retinal flat-mounts immunolabeled for GFAP at 4w after RI. **(F)** Quantification of GFAP immune-intensity by Image J. (One-way ANOVA, n = 5/group).

We further corroborated the finding with flow cytometry by characterizing the morphological differences of microglia and infiltrated macrophages after RI. Double-immunolabeling for Iba-1 and a retinal ganglion cell (RGC) marker Brn3a were performed to allow identification of microglia and macrophages in the ganglion cell layer (**Figure 4A**). We found that while most Iba-1^+^ cells in the control retinas displayed small cell bodies and ramified cell morphology, there was a large increase in Iba1^+^ cells (*P* < 0.001) and shorter dendrites (*P* = 0.003) in the retina with RI that was subjected to vehicle treatment (**Figures 4A–C**). Injection of CSF-1RAb reversed the morphological changes induced by RI (**Figures 4A–C**). As studies suggested that activated microglia trigger astrocyte activation and reactive gliosis to cause neuron loss (42), we next examined astrocyte morphology in the retina. Upregulation of glial fibrillary acidic protein (GFAP) is a hallmark of reactive astrocytes and commonly used to assess their responses. As expected, RI led to a significant increase of GFAP expression in the retina of vehicle-treated group (*P* < 0.001). Remarkably, CSF-1RAb treatment significantly attenuated RI-induced increase of GFAP immunolabeling (*P* = 0.002), as was confirmed by quantification of immune-intensity in flat-mount retinas (**Figures 4E, F**). Together, these results suggest that intravitreal injections of CSF-1RAb after RI effectively suppressed microglia/macrophage- and T cell-mediated responses without depleting the microglia and sustaining the homeostasis of retinal immune microenvironment.

### CSF-1RAb Treatment Improved RGC Survival and Visual Function

**Figure 5:**
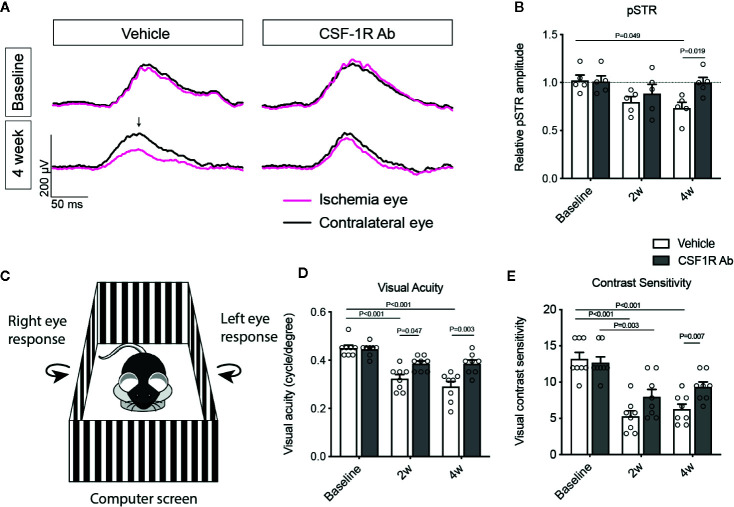
Therapeutic administration of CSF-1RAb improved RGC and visual functions in mice after RI. **(A)** Representative wave forms of pSTR in mice before (baseline) and at 4 weeks (4 week) RI, showing impaired RGC function in vehicle-treated control, but not in CSF-1RAb administered group post RI. **(B)** Relative pSTR amplitudes normalized to the contralateral uninjured eye (n = 5/group). **(C)** Schematic illustration of OMR-based assays. A mouse is placed on the platform in the middle, surrounded by four identical computer screens. Vertical sing-wave gratings are projected on the screens and rotated at a constant speed of 12^°^/s. **(D, E)** Visual acuity **(D)** and contrast sensitivity **(E)** assessed at 2 (2w) and 4 weeks (4w) post RI (Two-way ANOVA, n = 8/group).

To determine the impacts of CSF-1RAb on retinal neuron survival and function, we quantified RGCs in retinal flat-mounts immunolabeled for an RGC marker Brn3a (**Figure 4A**). Correlated with RI-induced increase in Iba-1^+^ cells, there was a significant loss of RGCs (*P* = 0.003) in the vehicle-treated retinas (**Figures 4A, D**). In contract, treatment of CSF-1RAb improved RGC survival after RI (**Figures 4A, D**). Next, we evaluated RGC responses by pSTR, the ERG component directly reflecting the functionality of RGCs (36). RI induced significant decreases in pSTR amplitudes as measured at 2 and 4 weeks post injury, when were compared to the baseline (**Figures 5A, B**), indicating an RI-induced functional impairment of RGCs. In agreement with the increased RGC survival, CSF-1RAb-treatment also significantly improved pSTR amplitudes at 4 weeks (*P* = 0.012) post RI compared to vehicle-treated group (**Figure 5B**). We observed no significant reduction of pSTR amplitudes in CSF-1RAb-treated group at 4 weeks post RI compared to non-injured control eyes. To address the paramount question of whether enhanced RGC survival and function confer to improvement in vision, we assessed mouse visual behavior using optomotor response (OMR)-based assay that measures visual acuity and contrast sensitivity (33). As significant reduction of visual acuity and contrast sensitivity was observed following RI, CSF-1RAb administration improved visual acuity and contrast sensitivity at both 2- and 4-weeks post RI as compared to vehicle control group (**Figures 5D, E**). These results indicate that weekly injection of CSF-1RAb improved RGC survival and visual function and thus presents a promising therapy after RI.

### CSF-1R Antibody Inhibited the Pro-Inflammatory Responses in Both Mouse and Human Microglia

**Figure 6:**
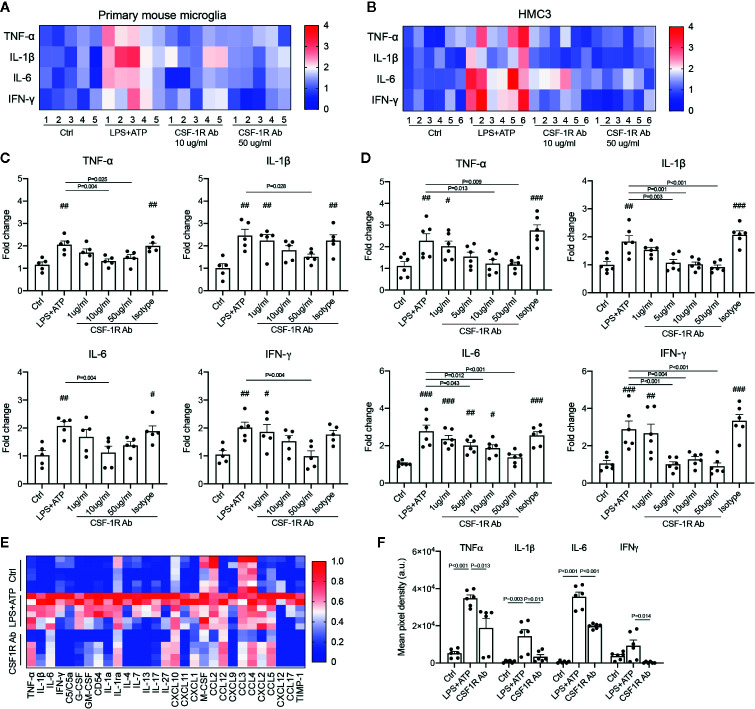
CSF-1RAb alleviated the expressions of inflammatory cytokines in both primary mouse microglia and human microglial cell line (HMC3). **(A, B)** Heatmaps of inflammatory cytokines in primary mouse microglia **(A)** and HMC3 **(B)** cultures 24 h after LPS (100 ng/ml) and ATP (100 μM) stimulation in the absence or presence of CSF-1RAb or isotype control. **(C, D)** Results of qPCR showing changes in levels of expression of TNF-α, IL-1β, IL-6, and IFN-γ in the primary mouse microglia **(C)** and HMC3 **(D)** cultures. **(E, F)** Heatmap of the cytokine arrays **(F)** and TNF-α, IL-1β, IL-6, and IFN-γ protein levels determined by proteome profiler mouse cytokine array kit taken from primary microglial cultures received no treatment (Ctrl), treated with LPS+ATP (LPS+ATP), or LPS+ATP and CSF-1RAb (CSF-1RAb).^#^ *P* < 0.05, ^##^ *P* < 0.01, ^###^ *P* < 0.001 compared to control (One-way ANOVA, n ≥ 5/group).

**Figure 7:**
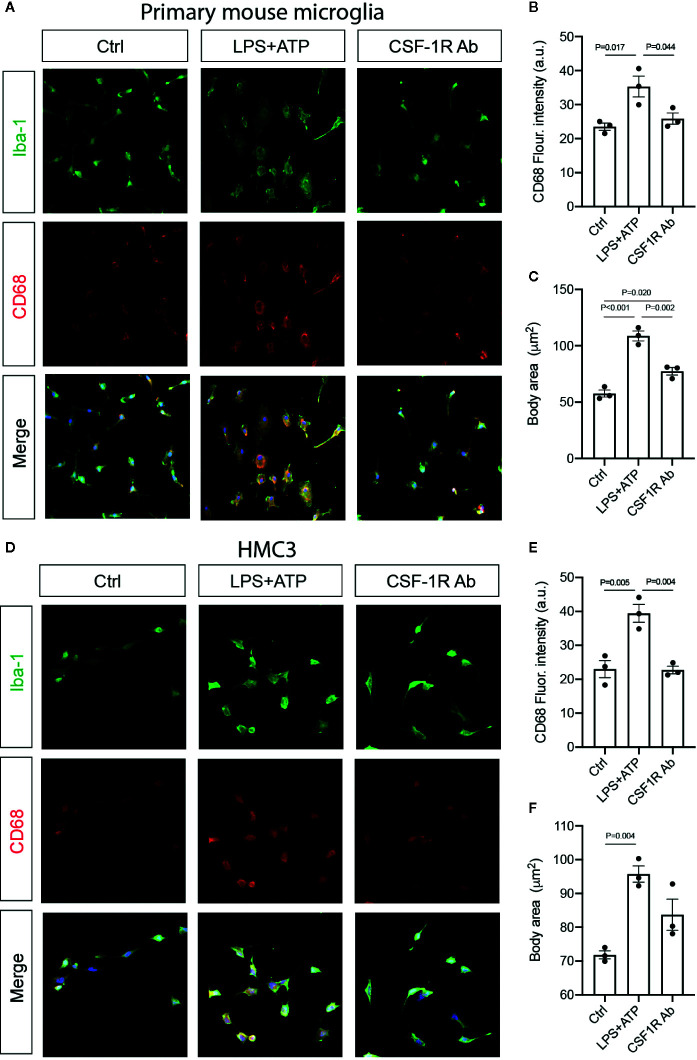
CSF-1RAb suppressed microglial activation in cultured primary mouse microglia and human microglial cell line HMC3. **(A–F)** Epifluorescence photomicrographs of primary mouse microglia **(A)** and HMC3 **(D)** cultures that were double-immunolabeled for Iba-1 (green) and CD68 (red) before (Ctrl) or at 24 h after LPS + ATP stimulation in the absence (LPS + ATP) or presence of CSF-1RAb (CSF-1R Ab), and quantification of CD68 fluorescence intensity **(B, E)** and cell body area **(C, F)** in primary mouse microglia **(B, C)** and HMC3 **(E, F)**. (One-way ANOVA, n = 3/group).

To determine if CSF-1RAb worked in human microglia, we compared its effects on the production of pro-inflammatory cytokines induced by potent inflammatory stimulators in a human microglial cell line, HMC3 (36), with that of primary mouse microglia. Mouse microglial cells were isolated as described above. Both primary microglia and HMC3 cells were stimulated with LPS (100 ng/ml) and ATP (100 μM) in the absence or presence of various concentrations of CSF-1RAb or an isotype control. qPCR detected significant increases in levels of expression of pro-inflammatory cytokines, TNF-α, IL-1β, IL-6, and IFN-γ, following LPS and ATP stimulation in both primary mouse microglia and HMC3 cells (**Figures 6A–D**). Addition of various concentrations of CSF-1RAb induced dose-dependent suppression of mRNA levels of pro-inflammatory cytokines in both mouse and human microglial cultures. The effect of CSF-1RAb on the suppression of inflammatory cytokine expression were verified by cytokine array analysis, which measures the protein levels of 32 most commonly studied cytokines. As LPS and ATP treatment in purified primary microglial cultures of mice induced the production of proinflammatory cytokines, addition of CSF-1RAb significantly reduced the levels of TNF-α, IL-1β, IL-6, and IFN-γ, as well as other 23 cytokines examined (**Figures 6E, F** and **Figure S2**). In contrast, addition of CSF-1RAb alone to control microglial cultures in the absence LPS and ATP stimulation showed no significant effect on proinflammatory cytokine expression (**Figure S3A**). Moreover, CSF-1R was detected only in microglia, but not other retinal cell types (**Figures S3B, C**), supporting a targeted focal effect of CSF-1RAb. Suppression of microglial activation by CSF-1RAb was further confirmed by immunolabeling for activated microglial marker CD68, showing the upregulation of CD68 expression in LPS-treated mouse and human microglia only in the absence of CSF-1R (**Figure 7**). Collectively, these results suggest that CSF-1R is a promising therapeutic target and have established the translational potential of CSF-1R inhibition for RI.

## Discussion

The present study demonstrated in both rat and mouse models that RI induced early microglial activation and macrophage infiltration, and subsequent recruitment of lymphocytes and CD4^+^ T cells. Therapeutic administration of CSF-1RAb weekly did not deplete microglia, but suppressed RI-induced glial activation and the associated changes of retinal immune microenvironment. Importantly, this resulted in improvement of RGC survival and visual function following RI. CSF-1RAb showed similar suppressive effect on inflammatory cytokine productions in cultured primary mouse microglia and human microglia cell line. Our results suggest CSF-1R as a promising therapeutic target and establish the translational potential of CSF-1RAb for currently untreatable condition of RI.

As both rats and mice are commonly used for the study of RI and other retinal diseases, comparison of data from both models are valuable by providing important verification in different animal species. Our results confirmed that acute RI induced inflammatory cytokines that were peaked day 1 post RI and microglial responses peaked at 7 days post-injury (**Figures 1**–**3**), and they gradually returned to the control levels by day 14 after injury in both species. They include the induction of TNF-α, IL-1β, IL-6 and INF-γ and other cytokines in 12–48 h after transient RI and lasted for a week, which observation has been extensively reported and verified in both the protein and mRNA levels (43–48). These reports are in agreement with our qPCR results in the present study. The suppression of the pro-inflammatory cytokines by administration of CSF-1R antagonist has also been documented (49). Importantly, weekly administration of CSF-1RAb in our study is sufficient to maintain the suppression of microglial, macrophage, and T cell activations beyond 14 days post injury.

Our previous studies suggested a critical role for retinal immune responses in the etiology of neurodegeneration associated with both acute RI-induced and chronic glaucomatous optic nerve damage (24, 25); the prolonged phase of the immune responses offers a therapeutic window for preserving vision after RI. The present research demonstrated an early onset of microglia/macrophage activation that proceeds lymphocyte and T cell infiltration and provides an attractive target for therapeutic interventions. This finding is in line with the reported increase of Iba-1^+^ cells in the ganglion cell layer between 1 and 14 days post RI (38) or optic nerve injury (50). Microglial activation is also found to underlie the cause of neuron loss in glaucoma and other neurodegenerative diseases (6, 51–53). It led to a pro-inflammatory retinal microenvironment and the disruption of blood-retina-barrier that enables the infiltration of lymphocytes and T cells (54, 55). It used to be thought that microglia and macrophages respond to inflammatory stimulators, such as LPS, to adopt a M1 phenotype and produce high levels of pro-inflammatory cytokines, but respond to IL-4 or T_H_2 cytokines to induce an “alternative” M2 phenotype to release anti-inflammatory mediators. Recent single RNA sequencing analysis, however, revealed that microglial activation is a dynamic process, involving multiple transcriptionally distinct subtypes, and it can present mixed characters between the M1/M2 phenotypes following injury (54, 56). Given the complex biological processes microglia involve, it is not surprising that complete removal of microglia is detrimental to neuronal survival (20, 21). Thus, it is imperative that administration of CSF-1RAb must be dosed to allow effective suppression of glial activation and the subsequent retinal immune responses without depleting microglia for RI treatment.

CSF-1R/CD115 is reported to be a key regulator for microglial survival, proliferation, differentiation, and function (57). Various CSF-1R antagonists have been developed, and they were shown to efficiently deplete microglia in mice (19, 58–60). Oral administration of CSF-1R antagonist PLX5622 for 14 consecutive days caused 95–99% depletion of microglia in the brain and retina (21, 59). Injection of CSF-1R antagonist AFS98 is sufficient to deplete microglia from the embryonic mouse forebrains (60). Injections of CSF-1R antibody for 2 consecutive days also depleted microglia from the embryonic forebrain that repopulate in 8 days (60). Despite that the depletion of microglia by CSF-1R antagonists is transient and cells repopulate the tissue after the withdrawal of the inhibitor, most often they lead to decreased survival of neurons, especially after injury (21, 59, 60).

Interestingly, studies that giving CSF-1R antagonist after the disease onset suppressed neurotoxic microglia and reduced neurodegeneration following traumatic brain injury (9), implicating a potential benefit of blocking CSF-1R signaling. We proposed that therapeutic administration of CSF-1RAb at a low dose or frequency may suppress microglial activation without depleting the cells. In the present study, we observed significant decrease of microglia 6 days after the first CSF-1R antibody injection. Weekly administration of CSF-1RAb was sufficient to suppress microglial activation until mice were sacrificed, suggesting that its effect lasts over 7 days. This is in line with the report that microglia began to repopulate the retina ~7 days after antibody withdrawal (59). In addition to microglia, macrophages which also expressed CD11b were found to follow a similar kinetic of suppression by CSF-1RAb administration (**Figures 3B–D**). Our results of flow cytometry and immunohistochemistry confirmed that weekly injection of CSF-1RAb intravitreally after RI attenuated the inflammatory responses without eliminating the microglia, and dampened lymphocyte and T cell infiltration and reactive gliosis, leading to neuroprotection and visual function recovery.

It is not surprising that visual contrast sensitivity was highly affected and more so than the visual acuity following RI. Visual contrast sensitivity is shown to be a sensitive measure that detects subtle defects or improvements in primarily retinal ganglion cell functions in human, especially early on in a disease process (61). This was also confirmed in rodent models of retinal neurodegenerative conditions, such as glaucoma, when the contrast sensitivity and visual acuity usually show a great consistency (33, 62). Although much remains to be learned about the factors that affect the visual contrast sensitivity, it is increasingly recognized that it may be used as a valuable reference for early detection of visual defect.

At last, we exploited the potential effect of CSF-1RAb on human microglia by comparing the responses with primary mouse microglia in culture. LPS is a potent inflammatory stimulator, especially when it works together with ATP on microglia. They induce inflammatory cytokines, such as TNF-α, IL-1β, IL-6, and IFN-γ. We observed similar dose-dependent responses between mouse and human microglia, showing reduced levels of inflammatory cytokines in responding to CSF-1RAb administration, suggestive of a therapeutic potential of CSF-1RAb on human microglia. We corroborated this finding with the cytokine array demonstrating that administration of CSF-1RAb effectively suppressed the induction of 26 inflammatory cytokines following LPS stimulation at the protein levels. Together, they strongly support an anti-inflammatory effect of CSF-1RAb acting directly through microglia.

As intravitreal injection is becoming a routine procedure in ophthalmic clinics, it should be noted that eye injection does lead to increased risk of eye infection. However, it carries the advantage of allowing direct drug delivery to the retina and reducing off-target or adverse effects such as that caused by systemic exposure. CSF-1R is reported to be expressed by neurons in the hippocampus and cortex (63). Thus, oral administration of CSF-1R antagonists not only affects microglial activity in the brain, but also induces dysregulated neuronal calcium and synaptic responses (20, 64). Intravitreal injection of CSF-1RAb limits these undesirable side effects and enables targeted therapy through manipulating the local retinal microenvironment.

To the best of our knowledge, the present study provided novel evidence by targeting retinal immune microenvironment *via* intravitreal injection of CSF-1RAb and demonstrated not only RGC survival, but importantly, improved vision after RI. These findings have important clinical implications, especially given that currently no treatment is available for such a devastating condition of RI in clinic. It should be noted that there are limitations in the present study. Much still remains to be elucidated to fully uncover the mechanisms and signaling events underlying the effects of CSF-1R administration and the interactions between microglia and other immune cells following RI. Long-term outcome measures and safety profiles of CSF-1RAb are also needed before clinical trials are considered. Nevertheless, the present study uncovered a new therapeutic strategy for targeting retinal immune microenvironment. Rather than depleting microglia, by controlling their responses toward injury has the potential to prevent neuron loss and rescue vision in ischemic optic neuropathy.

## Data Availability Statement

All datasets presented in this study are included in the article/**Supplementary Material**.

## Ethics Statement

The animal study was reviewed and approved by the Animal Care Committee of Schepens Eye Research Institute of Massachusetts Eye and Ear, and the Eye and ENT Hospital of Fudan University.

## Author Contributions

DC, XS, and YT conceived and designed the study, and YT, ZX, LP, DZ, and LS performed the experiments and collected the data. YT, ZX, DZ, and LS performed the animal studies in rats. YT and LP performed the animal experiments in mice. YT and XC performed the cell culture experiments. YT, DC, JW, and K-SC, KR and GT analyzed the data. YT and DC wrote, revised, and finalized the manuscript. DC, K-SC and XS received grants and provided financial support to the study. All authors contributed to the article and approved the submitted version.

## Funding

This work was supported in part by grants from the National Institutes of Health (NIH)/National Eye Institute (NEI) (Grants EY025913 and EY025259 to DC), Massachusetts Eye and Ear Summit Fund to DC, the Core Grant for Vision Research from NIH/NEI to the Schepens Eye Research Institute (P30EY03790), the Bright Focus Foundation to K-SC, and the Major Program of National Natural Science Foundation of China (Grant 81790641 to XS).

## Conflict of Interest

DC and K-SC are co-inventors on the patent application for targeting CSF-1/CSF-1R to treat optic neuropathy.

The remaining authors declare that the research was conducted in the absence of any commercial or financial relationships that could be construed as a potential conflict of interest.

## Abbreviations

APC, antigen presenting cells; BRB, blood retina barrier; CSF-1R, colony-stimulating factor-1 receptor; CSF-1RAb, colony-stimulating factor-1 receptor antibody; CS, contrast sensitivity; ERG, electroretinogram; MHC-II, major histocompatibility complex; OMR, optomotor response/reflex; pSTR, positive scotopic threshold response; RGC, retinal ganglion cells; RI, retinal ischemic/reperfusion injury; VA, visual acuity.
