# Supplementary material for: Therapeutic Targeting of Retinal Immune Microenvironment With CSF-1 Receptor Antibody Promotes Visual Function Recovery After Ischemic Optic Neuropathy
Source: Front Immunol. 2020 Nov 13;11:585918. doi: 10.3389/fimmu.2020.585918 (PMC7691249; doi:10.3389/fimmu.2020.585918)
Supplement: Supplementary file 1 [file Table_1.docx]

Supplementary material

**Therapeutic targeting of retinal immune microenvironment with CSF-1 Receptor antibody promotes visual function recovery after ischemic optic neuropathy**

Yizhen Tang^1,2,3,4^, Zebin Xiao^5^, Pan Li^2,6^, Dongli Zhuang^1,3,4^, Kin-Sang Cho^2^, Kyle Robert^2^, Xiaoxiao Chen^1,3,4^, Lian Shu^1,3,4^, Guangxian Tang^7^, Jihong Wu^1,3,4^, Xinghuai Sun^1,3,4*^ and Dong F. Chen^2*^

^1^Eye Institute and Department of Ophthalmology, Eye & ENT Hospital, Fudan University, Shanghai, 200031, China

^2^Schepens Eye Research Institute of Massachusetts Eye and Ear, Department of Ophthalmology, Harvard Medical School, Boston, Massachusetts, USA

^3^NHC Key Laboratory of Myopia (Fudan University); Key Laboratory of Myopia, Chinese Academy of Medical Sciences, Shanghai, 200031, China

^4^Shanghai Key Laboratory of Visual Impairment and Restoration, Shanghai, 200031, China

^5^Department of Radiology, Eye & ENT Hospital, Fudan University, Shanghai, 200031, China

^6^School of Optometry, the Hong Kong Polytechnic University, Hong Kong, China

^7^Department of Ophthalmology, Shijiazhuang No. 1 Hospital, Hebei, China

***Correspondence:**
Co-corresponding author, email: [dongfeng_chen@meei.harvard.edu (D.F](mailto:dongfeng_chen@meei.harvard.edu%20(D.F). Chen), xhsun@shmu.edu.cn (X. Sun).

Supplementary Caption:

1. Table S1. Sequences of primers used for qPCR.
2. Figure S1. Retinal ischemia/reperfusion injury induced retinal inflammation.
3. Figure S2. CSF-1RAb alleviated the expressions of inflammatory cytokines in primary mouse microglia.
4. Figure S3. Selective expression of CSF-1R in microglia and the effect of CSF-1RAb in purified microglial cultures.

Table S1. Sequences of primers used for qPCR

| Species | Genes | Forward 5’-3’ | Reverse 5’-3’ |
| --- | --- | --- | --- |
| Rat | Gapdh | TTCAACGGCACAGTCAAGG | CACCAGTGGATGCAGGGAT |
|  | CD16 | AACGGCACTGCTACTTACGG | CGAGATGAGGCTTTTGTTATGG |
|  | CD86 | TAGGGATAACCAGGCTCTAC | CGTGGGTGTCTTTTGCTGTA |
|  | CD206 | CTCTAAGCGCCATCTCCGTT | ATGATCTGCGACTCCGACAC |
|  | TNF-α | CGTCGTAGCAAACCACCAAGC | CCAGTCGCCTCACAGAGCAAT |
|  | IL-1β | AATGACCTGTTCTTTGAGGCTGAC | CGAGATGCTGCTGTGAGATTTGAAG |
|  | TGF-β | CCGCAACAACGCAATCTATG | AGCCCTGTATTCCGTCTCCTT |
| Mouse | Β-actin | GGCTGTATTCCCCTCCATCG | CCAGTTGGTAACAATGCCATGT |
|  | TNF-α | CAAAGGGATGAGAAGTTC | AGATGATCTGAGTGTGAG |
|  | IL-1β | CCTCAATGGACAGAATATC | TTACACAGGACAGGTATAG |
|  | IL-6 | ACCTGTCTATACCACTTCA | GCATCATCGTTGTTCATAC |
|  | IFN-γ | ACACCTGATTACTACCTTCTTC | GACCTCAAACTTGGCAATAC |
| Human | Gapdh | GTCTCCTCTGACTTCAACAGCG | ACCACCCTGTTGCTGTAGCCAA |
|  | TNF-α | CTCTTCTGCCTGCTGCACTTTG | ATGGGCTACAGGCTTGTCACTC |
|  | IL-1β | CCACAGACCTTCCAGGAGAATG | GTGCAGTTCAGTGATCGTACAGG |
|  | IL-6 | TTCTGCCAGTGCCTCTTTGCTG | AGACAGCCACTCACCTCTTCAG |
|  | IFN-γ | GAGTGTGGAGACCATCAAGGAAG | TGCTTTGCGTTGGACATTCAAGTC |


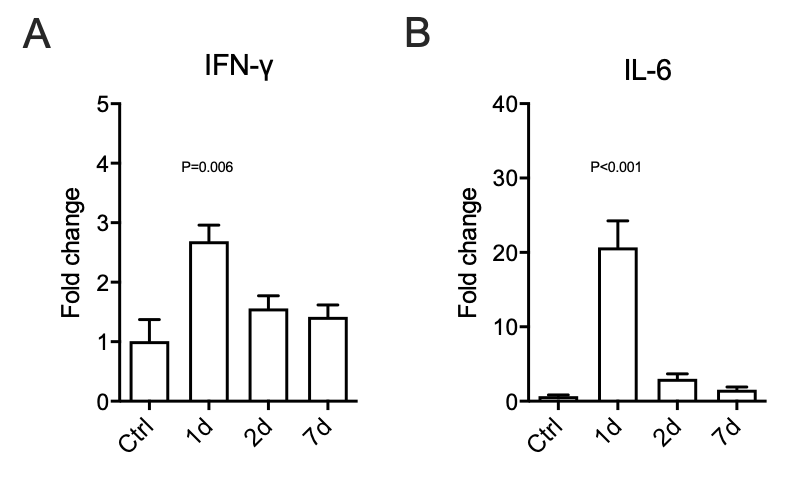


Figure S1. Retinal ischemia/reperfusion injury induced IFN-γ and IL-6 upregulation. Results of qPCR showing fold changes of IFN-γ (A) and IL-6 (B) mRNA levels in retinas of control mice (Ctrl) and mice at 1 – 7 days (d) post RI. One-way ANOVA compared to control (n = 3/group).


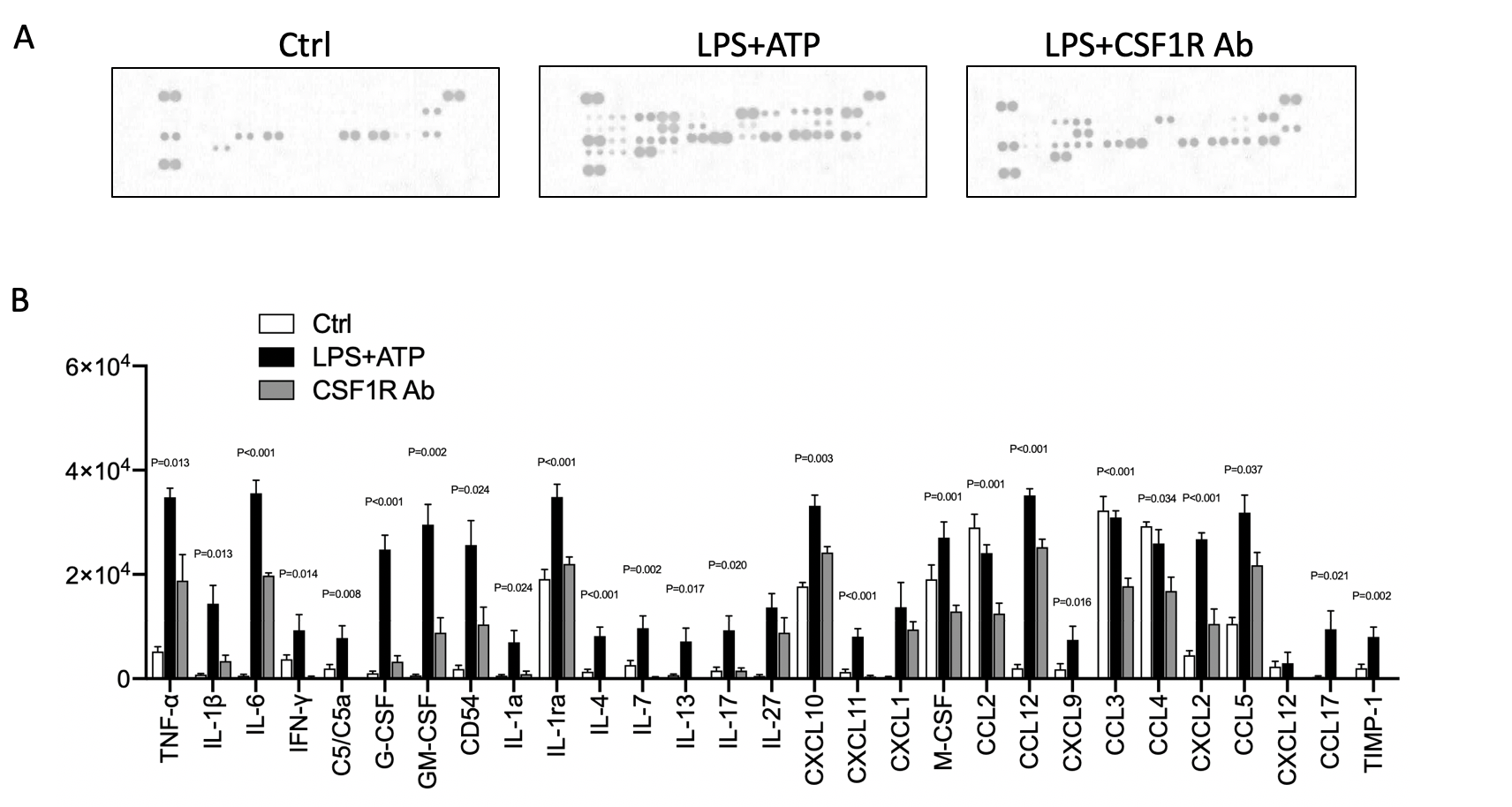


Figure S2. CSF-1RAb administration alleviated the expression of inflammatory cytokines in primary microglial cultures of mice. (A-B) Representative images of cytokine array membrane (A) and quantification (B) of inflammatory cytokines using proteome profiler cytokine array in primary mouse microglial cultures treated with LPS + ATP or LPS + ATP + CSF-1RAb (10 μg/ml). One-way ANOVA comparing between LPS + ATP and CSF-1RAb treatment groups (n = 6/group).


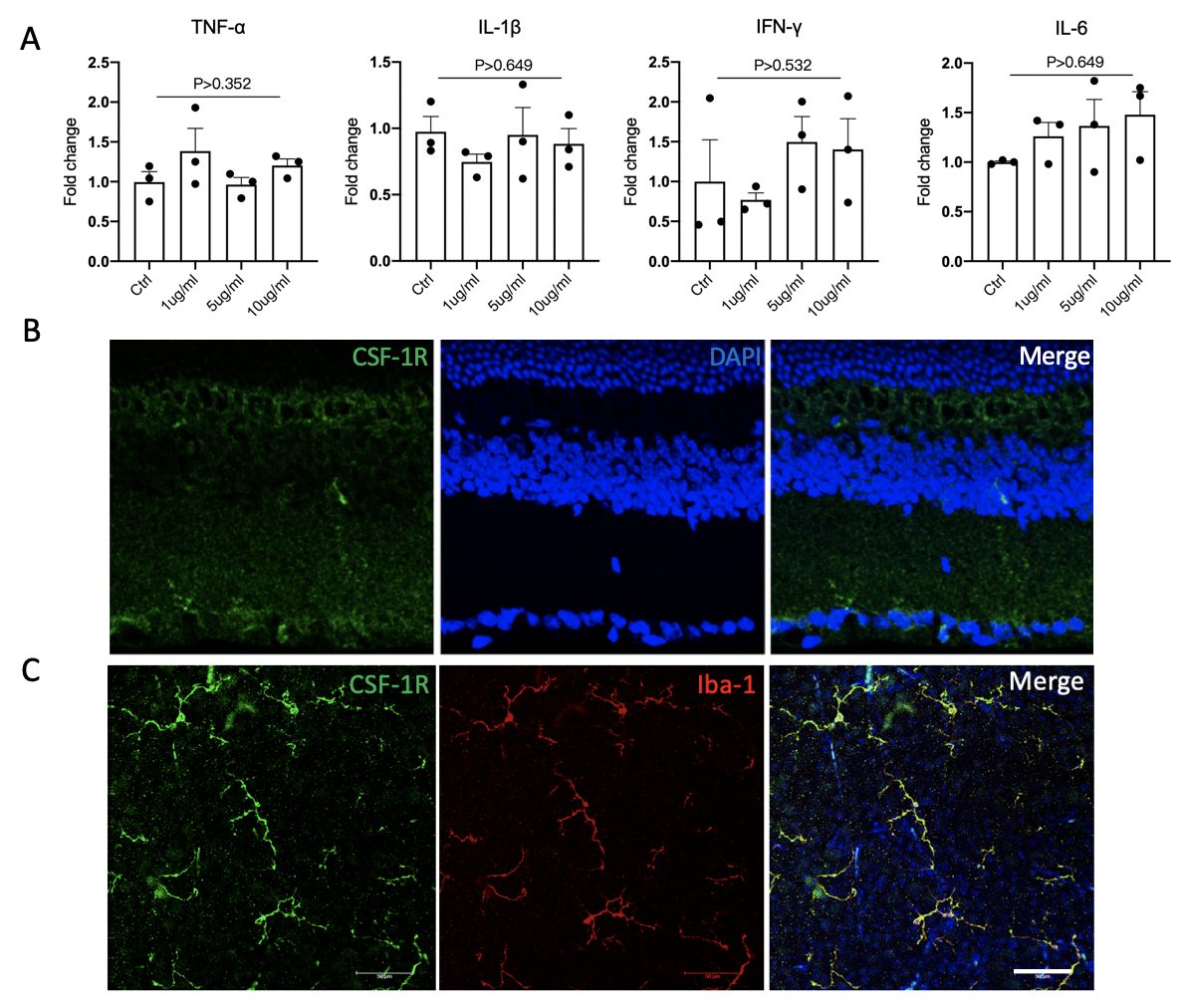


Figure S3. Selective expression of CSF-1R in microglia and the effect of CSF-1RAb in purified microglial cultures. (A) Results of qPCR showing no significant fold changes (of TNF-a, IL-1β, IFN-γ, and IL-6 mRNA levels in primary mouse microglia treated with CSF-1RAb at 1, 5 and 10 μg/ml, respectively (One-way ANOVA, n = 3/group). (B) Epifluorescence photomicrographs of retinal sections of adult mice that were immunolabeled for CSF-1R (green) and counter-stained with a nuclear marker DAPI (blue). (C) Photomicrograph of retinal flat-mount that were double-immunolabeled for CSF-1R (green) and microglial marker Iba-1 (red). Note that CSF-1R signal was strongly colocalized with Iba-1 immunolabeling, but was scarce in other retinal layers or areas. Scale bar: 50 μm.
